# The Rare Co-occurrence of Clostridioides difficile Infection and Pseudomonas Meningitis in an Infant: A Case Report

**DOI:** 10.7759/cureus.87103

**Published:** 2025-07-01

**Authors:** Ia Khurtsilava, Darejan Kanjaradze, Ekaterine Gozalishvili, Tamar Gachechiladze, Teimuraz Mikeladze

**Affiliations:** 1 Department of Pediatrics, Tbilisi Pediatric Private Clinic, Tbilisi, GEO; 2 Department of Pediatrics, Tbilisi Medical Academy, Tbilisi, GEO; 3 Department of Pediatric Intensive Care Unit, Tbilisi Pediatric Private Clinic, Tbilisi, GEO; 4 Pediatric Emergency Department, Tbilisi Pediatric Private Clinic, Tbilisi, GEO; 5 Neurology, MediClub Georgia, Tbilisi, GEO; 6 Department of Neurology, Tbilisi Pediatric Private Clinic, Tbilisi, GEO; 7 Department of Pediatrics, University of Georgia, Tbilisi, GEO; 8 Department of Pediatrics, European University, Tbilisi, GEO

**Keywords:** clostridioides difficile infection, gram negative meningitis, pediatric gastroenterology, pediatric infectious disease, pseudomonas aeruginosa meningitis

## Abstract

This case report presents a five-month-old infant who developed clinically manifested Clostridioides difficile (C. difficile) infection (CDI) and Pseudomonas aeruginosa (P. aeruginosa) meningitis. Initially diagnosed with pseudomembranous enterocolitis and treated with oral vancomycin, the child showed gastrointestinal improvement but continued to experience persistent fever, prompting further evaluation. Neuroimaging revealed ventriculomegaly, leading to a lumbar puncture that confirmed bacterial meningitis. Empiric antibiotics were initiated, and amikacin was added following CSF culture results that identified P. aeruginosa, resulting in significant clinical improvement. This report highlights the diagnostic challenges of P. aeruginosa meningitis in infants, particularly when complicated by secondary infections like CDI. Our objective is to raise awareness about the fact that although P. aeruginosa meningitis is extremely rare in the pediatric population and CDI is infrequently symptomatic in infants, early recognition and aggressive treatment can improve outcomes and reduce complications.

## Introduction

Bacterial meningitis is a severe and potentially fatal infection characterized by inflammation of the meninges, the protective membranes surrounding the brain and spinal cord [1]. It is a medical emergency requiring prompt diagnosis and immediate treatment to minimize morbidity and mortality. The causative bacterial pathogens vary by age group, with Group B Streptococcus being the most common in infants younger than two months, while Streptococcus pneumoniae is the leading cause in most other age groups. Among individuals aged 11 to 17 years, Neisseria meningitidis is the most frequently identified pathogen [1,2].

Gram-negative bacilli such as Klebsiella spp., Escherichia coli (E. coli), Enterobacter spp., and Pseudomonas aeruginosa (P. aeruginosa) are less common, with E. coli and P. aeruginosa being the most prevalent among them [1,2]. Nosocomial bacterial meningitis often results from invasive procedures, traumatic head injuries, and intraventricular catheters (IVS). Though rare, they are often associated with serious conditions and risk of complications, and high mortality rates. Diagnosis can be challenging, especially in small children, due to the absence of classical neuroinfection symptoms, leading to potential delays in treatment and worsening patient outcomes. Managing P. aeruginosa meningitis is also difficult due to the limited number of effective antimicrobial agents and their poor penetration through the blood-brain barrier.

Clostridioides difficile (C. difficile) infection (CDI) has traditionally been associated with adult and elderly populations, particularly those with recent antibiotic exposure or hospitalization. However, there is growing evidence that suggests that CDI is also a significant concern in pediatric patients, including young infants and children. While asymptomatic colonization with C. difficile is common in neonates, distinguishing between colonization and true infection remains a challenge [3,4]. This case report describes an infant who developed clinically manifested CDI, was successfully treated, and was later diagnosed with P. aeruginosa meningitis.

## Case presentation

A five-month-old severely dehydrated child was brought to the pediatric emergency department. The mother reported that over the past few days, the child had experienced countless episodes of diarrhea, and their pediatrician had prescribed oral vancomycin (60 mg/day orally) after confirming the presence of C. difficile toxin. On examination, the child had bluish/purplish skin discoloration, dry and scaly mucous membranes, weak peripheral pulses, decreased skin turgor and elasticity, and a mildly sunken anterior fontanelle. The Pediatric Glasgow Coma Scale (PGCS) score was 8. The vital signs were as follows: heart rate (HR): 174 bpm; respiratory rate (RR): 62 breaths per minute; SpO₂: 81%; blood pressure (BP): 47/28 mmHg; capillary refill time (CRT): six seconds; temperature (T): 38.4°C; and weight: 4800 grams.

The child’s condition was assessed as hypovolemic shock, and immediate fluid resuscitation was initiated. Initial blood gas and electrolyte analysis revealed severe acidemia and significant electrolyte imbalances (venous PH: 6.9, NA: -168, K: 3.5, Cl: 127). Despite multiple fluid boluses, the patient remained hemodynamically unstable, prompting the initiation of a dobutamine infusion, and the patient was switched to mechanical ventilation. Initial laboratory tests were performed on the day of admission and the next morning (Tables 1, 2). History revealed that over the past three months, the patient had experienced multiple episodes of fever requiring hospitalization with various diagnoses, including pertussis, bronchiolitis, and sepsis. During these hospital stays, broad-spectrum antibiotics were administered. Four days ago, the family had deliberately left the pediatric clinic and consulted their pediatrician, who ordered a C. difficile toxin test. The patient was subsequently diagnosed with pseudomembranous enterocolitis.

**Table 1:** Biochemical and hormonal profile ALT: alanine transaminase; AST: aspartate transaminase; CRP: C-reactive protein

Test	Result	Reference range
Morning cortisol, nmol/L	1152	171–536
ALT, U/L	36.3	<60
AST, U/L	45.7	<84 U/L
Creatinine, µmol/L	34.2	<80
CRP, mg/L	22.8	<6
17-OH progesterone, ng/mL	4.69	1.8-20.0
Total protein, g/L	52.0	66–87
Albumin, g/L	34.0	35–52

**Table 2:** CBC results CBC: complete blood count; Hb: hemoglobin; HCT: hematocrit; MCV: mean corpuscular volume; PLT: platelets; RBC: red blood cells; RDW-CV: red blood cell distribution width-coefficient of variation; RDW-SD: red blood cell distribution width-standard deviation; WBC: white blood cells

Initial CBC		CBC before discharge	
Parameter	Result	Result	Reference range
Hb, g/L	91	102	110–140
RBC, × 10¹²/L	3.5	3.9	4.1–5.5
PLT, × 10⁹/L	544	339	150–450
WBC, × 10⁹/L	9.3	6.8	5.0–15.0
Granulocytes, %	54.4	26.3	~50–70
Agranulocytes, %	41.8	63.3	~30–50
Monocytes, %	3.8	10.4	2–10
HCT, %	28.1	32.2	33–39
MCV, fL	79.7	82.3	77–95
RDW-CV, %	14.4%	15.6	11.5–14.5
RDW-SD, fL	46.7	52.0	37–54

The day of admission to our clinic marked the 14th day of persistent fever and diarrhea. No blood or mucus was present in the stool during admission. Blood cultures were negative, and no evidence of systemic bacterial infection or endocrine causes of the electrolyte imbalance was found. Neurological infection remained a concern despite the absence of classical signs and symptoms. Initially, lumbar puncture was deferred due to the patient's hemodynamic instability, and subsequently, the parents repeatedly refused to give consent for the procedure. There was some clinical improvement, the stool pattern normalized, and the child began to eat adequately; however, the patient’s condition remained concerning. Episodes of fever persisted. Oral vancomycin was discontinued.

**Figure 1:**
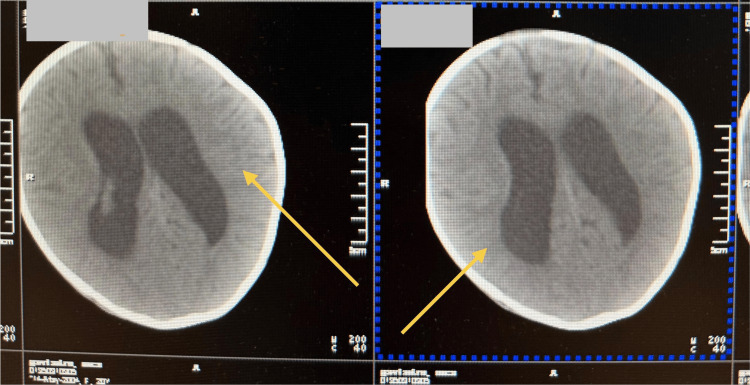
CT of the head showing ventriculomegaly CT: computed tomography

Neuroimaging (neurosonoscopy) revealed ventriculomegaly (anterior horn width D: 14.07 mm, S: 12.84 mm). CT scan of the head also documented ventriculomegaly (Figure 1). After several conversations, the parents ultimately agreed to give their consent for a lumbar puncture (Table 3). The procedure confirmed bacterial meningitis. Empirical antibiotic treatment for meningitis was initiated with vancomycin (60 mg/kg/day) and meropenem (40 mg/kg/day). CSF culture grew P. aeruginosa 10^5 CFU/ml. Amikacin (21 mg/kg/day) was added to the treatment regimen.

**Table 3:** CSF analysis results CSF: cerebrospinal fluid; RBC: red blood cells; WBC: white blood cells

Initial CSF fluid		CSF fluid result after treatment	
Parameter	Result	Result	Reference range
Color	Clear	Clear	Clear
Specific gravity	1.015	1.015	1.006–1.007
pH	7.5	8.5	7.35–7.40
Protein, g/L	13.1	1.45	0.22–0.33
Glucose, mmol/L	0.863	1.49	2.8–3.9
Cytosis	1194	5	<32
Erythrocytes (RBCs), cells/µL	10-12	1–2	0–5
Leukocytes (WBCs), cells/µL	High	8–9	0–5

Antibiotic therapy was continued for 21 days, and a repeat lumbar puncture showed no abnormalities and no bacterial growth. During the antibiotic course, the patient became afebrile within 48 hours and showed marked clinical improvement. The patient was discharged with a follow-up visit scheduled in two weeks. At the follow-up visit, the child had no complaints, was feeding well, with normal stool and urination patterns. No neurological deficits were observed, though a mild developmental delay was noted. Four months after discharge, the parents contacted us and informed us that minimally invasive neurosurgery had been performed due to hydrocephalus. Otherwise, the child is doing well, with no significant complaints. The child is developing normally, gaining weight adequately, and has no neurological deficits.

## Discussion

CDI in infants presents a diagnostic and therapeutic challenge due to the high rates of asymptomatic colonization in infants and the immature gut receptor expression, which may confer reduced sensitivity to C. difficile toxins [3,4]. We discussed a case of a five-month-old infant who developed severe diarrhea and dehydration following previous hospitalizations and multiple courses of broad-spectrum antibiotics, eventually being diagnosed with pseudomembranous enterocolitis. This aligns with the findings of Li et al., who reported a similar pattern in a one-month-old infant developing symptomatic CDI after extensive antibiotic exposure [3].

CDI in infants is often overlooked or misattributed to other causes due to its rare symptomatic manifestation and overlapping clinical signs with other gastrointestinal disorders. However, the presence of persistent diarrhea, clinical deterioration, and a positive C. difficile toxin assay in our patient supported the diagnosis of true infection rather than colonization. As in the case report by Tibesar, our patient was diagnosed with pseudomembranous colitis, supporting the concept that CDI, while rare, can indeed manifest in neonates and infants and should not be dismissed solely due to age [4]. Further complicating the clinical picture, the patient in this case also developed P. aeruginosa meningitis. P. aeruginosa meningitis in pediatric patients is exceedingly rare, as outlined by Cotran-Lenrow et al., who reported a case of community-acquired infection in a child without prior neurosurgical intervention [1,2]. Although the exact origin in our case remains unidentified, the possibility of nosocomial colonization followed by hematogenous spread cannot be excluded, especially in the setting of prolonged antibiotic therapy and compromised mucosal barriers.

Diagnosis of bacterial meningitis in infants is particularly challenging due to nonspecific symptoms and the difficulty in performing lumbar punctures in critically ill patients. In our case, diagnostic delay occurred due to both clinical instability and parental hesitancy regarding invasive procedures. Nonetheless, neuroimaging findings prompted re-evaluation, and CSF culture eventually confirmed P. aeruginosa as the causative pathogen. This shows the importance of staying alert and being persistent in diagnostic efforts, even when the usual signs of meningitis are absent. Management of P. aeruginosa meningitis is complicated by the pathogen’s inherent resistance to many antibiotics and poor CNS penetration of most effective agents. The successful use of amikacin in our case contributed to clinical recovery, emphasizing the importance of targeted therapy based on culture and sensitivity results. The resolution of infection and favorable short-term outcome following a 21-day antibiotic course further highlight the importance of individualized, aggressive management strategies.

## Conclusions

This report emphasizes the rarity and diagnostic challenges associated with CDI and P. aeruginosa meningitis in infants, particularly in those with prior antibiotic exposure and repeated hospitalizations. While CDI is typically associated with adults, infants can develop clinically significant infections, despite often having asymptomatic colonization. Furthermore, although extremely rare, infants may also develop meningitis caused by uncommon pathogens. Lastly, the report highlights the importance of prudent antibiotic use in pediatric populations to prevent both CDI and nosocomial infections like those related to P. aeruginosa.
